# Supplementary material for: Gold-Standard Chemical Database 137 (GSCDB137): A Diverse Set of Accurate Energy Differences for Assessing and Developing Density Functionals
Source: J Chem Theory Comput. 2025 Dec 1;21(24):12601–21. doi: 10.1021/acs.jctc.5c01380 (PMC12746454; doi:10.1021/acs.jctc.5c01380)
Supplement: Supplementary file 1 [file ct5c01380_si_001.pdf]

Supporting Information: Gold-Standard Chemical Database 137 (GSCDB137): A Diverse Set of Accurate Energy Differences for Assessing and Developing Density Functionals

Jiashu Liang[†] and Martin Head-Gordon[*,†,‡]

† *Kenneth S. Pitzer Center for Theoretical Chemistry, Department of Chemistry, University of California at Berkeley, Berkeley, California 94720, United States*

‡ *Chemical Sciences Division, Lawrence Berkeley National Laboratory, Berkeley, California 94720, United States*

E-mail: mhg@cchem.berkeley.edu

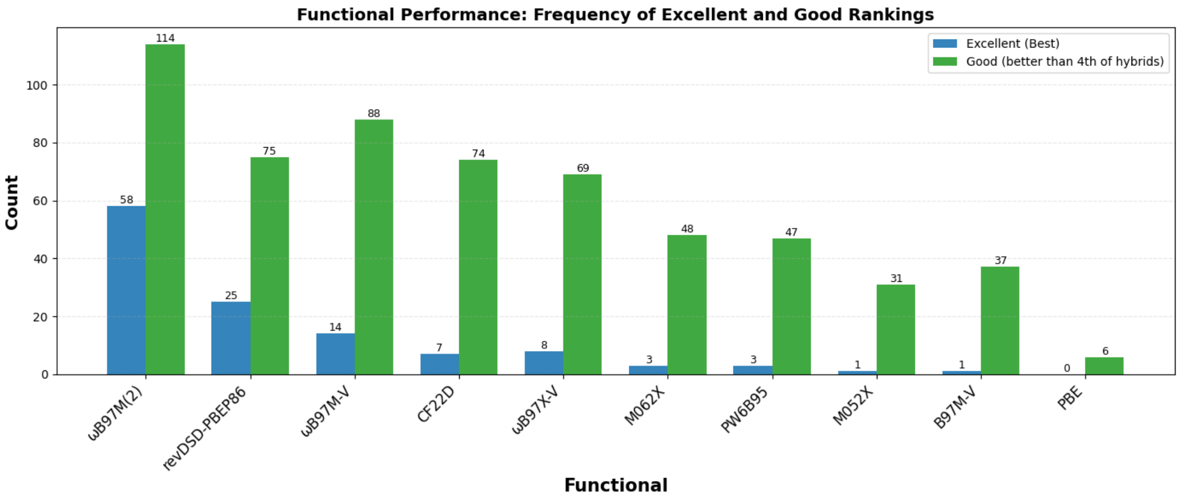

Figure S1: Histogram summarizing functional performance across all benchmark data sets. For each data set, the functional with the lowest error (defined in Section 4.1) contributes one *Excellent* count, while a functional contributes a *Good* count if its error does not exceed that of the fourth-best hybrid functional for the same data set. Displayed are all double hybrids (DHs), the six hybrids with the highest Good counts, and the top-performing meta-GGA and GGA by Good count. Excellent counts indicate peak accuracy, whereas Good counts reflect the breadth of consistently high performance. For brevity, dispersion-correction labels are omitted.

Table S1: Spin contamination analysis for molecules identified as "Difficult". The expected and κ-OOMP2 $\langle S^2 \rangle$ values are reported to assess the presence and degree of spin symmetry breaking.

Molecule label name	input S	Expected $\langle S^2 \rangle$	κOOMP2 $\langle S^2 \rangle$
Reference evaluated only at the restricted CCSD(T) level			
16_Rxn6_TS_0cat_WCPT27	0.0	0.0	0.37442
48_Isomer38_Styrene45	0.0	0.0	0.19079
BH9_05_10TS	0.0	0.0	0.25343
BH9_05_12TS	0.0	0.0	0.23487
BH9_05_15TS	0.0	0.0	0.21359
BH9_05_28TS	0.0	0.0	0.13999
BH9_05_29TS	0.0	0.0	0.10417
BH9_05_37TS	0.0	0.0	0.27310
C60ISO_6	0.0	0.0	0.45364
C60ISO_8	0.0	0.0	1.00544
CARBHB12_1CL	0.0	0.0	0.46411
CARBHB12_1CL_B	0.0	0.0	0.63986
CARBHB12_1N	0.0	0.0	0.56707
CARBHB12_1N_B	0.0	0.0	0.60702
CARBHB12_1O	0.0	0.0	0.51133
CARBHB12_1O_B	0.0	0.0	0.61909
CARBHB12_2CL_B	0.0	0.0	0.03752
CUAGAU2_M025_Cu6-i0	0.0	0.0	1.04834
CUAGAU2_M026_Ag6-i0	0.0	0.0	1.03938
CUAGAU2_M027_Au6-i0	0.0	0.0	1.04197
CUAGAU2_M028_Cu3-c1-i0	0.0	0.0	0.78269
CUAGAU2_M029_Ag3-c1-i0	0.0	0.0	0.71083
CUAGAU2_M030_Au3-c1-i0	0.0	0.0	0.76392
CUAGAU2_M043_Cu6-c2-i0	0.0	0.0	1.04588
CUAGAU2_M044_Ag6-c2-i0	0.0	0.0	1.04020
CUAGAU2_M045_Ag6-c2-i0	0.0	0.0	1.04070
CUAGAU2_M053_Ag6-i1	0.0	0.0	1.03336

Continuation of Table: Spin Contamination Analysis			
Molecule label name	input S	Expected S^2	kOOMP2 S^2
CUAGAU2_M054_Au6-i1	0.0	0.0	1.03795
CUAGAU2_M058_Cu5-c1-i1	0.0	0.0	1.07203
CUAGAU2_M059_Ag5-c1-i1	0.0	0.0	1.05372
CUAGAU2_M060_Au5-c1-i1	0.0	0.0	1.05964
CUAGAU2_M068_Ag6-c2-i1	0.0	0.0	1.05610
CUAGAU2_M079_Cu4-c2-O-i0	0.0	0.0	1.01259
CUAGAU2_M080_Ag4-c2-O-i0	0.0	0.0	1.00826
CUAGAU2_M118_Cu6-c2-NH3	0.0	0.0	1.04375
CUAGAU2_M120_Au6-c2-NH3	0.0	0.0	1.04089
CUAGAU2_M124_Cu6-c2-CO	0.0	0.0	1.04437
CUAGAU2_M125_Ag6-c2-CO	0.0	0.0	1.03938
CUAGAU_Ag03C1_LIN	0.0	0.0	0.71083
CUAGAU_Au03C1_LIN	0.0	0.0	0.76392
CUAGAU_Cu03C1_LIN	0.0	0.0	0.78269
DIPCS10_h2s_2+	0.0	0.0	0.15004
G2RC_6	0.0	0.0	0.58702
INV24_PCl3_TS	0.0	0.0	1.00683
ISOL24_i11p	0.0	0.0	0.73205
MX35_1208_MgO_01	0.0	0.0	1.00613
MME55_CDO_1C	0.0	0.0	0.21856
MME55_Hc_1_1	0.0	0.0	1.00476
MME55_Hc_1_2a	0.0	0.0	0.27781
Reference evaluated at the unrestricted CCSD(T) level			
Dip152_CH2-s2+	0.0	0.0	0.59668
Dip152_CH2-s2-	0.0	0.0	0.59765
Dip152_HNS1+	0.0	0.0	0.31640
Dip152_HNS1-	0.0	0.0	0.31648
Pol132_HNS	0.0	0.0	0.31644

Continuation of Table: Spin Contamination Analysis			
Molecule label name	input S	Expected S^2	kOOMP2 S^2
Pol132_HNS0+	0.0	0.0	0.36059
Pol132_HNS0-	0.0	0.0	0.27974
Pol132_HNS1+	0.0	0.0	0.30855
Pol132_HNS1-	0.0	0.0	0.31659
Pol132_HNS2+	0.0	0.0	0.31882
Pol132_HNS2-	0.0	0.0	0.31882
Reference evaluated beyond CCSD(T) level or with experimental references			
3d4dIPSS_Y_GS+	0.0	0.0	0.15539
ALKBDE10_mgo	0.0	0.0	1.00610
DAPD_Pd2	0.0	0.0	0.86952
IP23_BN	0.5	0.75	1.43082
IP23_BN_n	0.0	0.0	1.02580
IP23_C2	0.5	0.75	1.69754
IP23_C2_n	0.0	0.0	0.89473
ORBH36_CO3B_S01	0.0	0.0	0.57909
ORBH36_CO3B_S03T	0.0	0.0	0.86465
W4-17_b2	1.0	2.0	2.81628
W4-17_bn	0.0	0.0	1.02634
W4-17_c2	0.0	0.0	0.87228
W4-17_ch2-sing	0.0	0.0	0.59060
W4-17_cloo	0.5	0.75	1.25227
W4-17_s3	0.0	0.0	0.12031
W4-17_s4-c2v	0.0	0.0	0.75903
End of Table			
